# Carcinoma of Unknown Primary Origin Presenting as Rapidly Progressive Multifocal Skeletal Lesions and Fatal Systemic Thrombosis in a Dog: A Case Report

**DOI:** 10.3390/vetsci13090918

**Published:** 2026-09-07

**Authors:** Bumgyu Shin, Duhwan Park, Suhyun Lee, Jihoon Kim, Su-Hyung Lee, Minji Won, Hwi-Yool Kim

**Affiliations:** 1 Department of Veterinary Surgery, College of Veterinary Medicine, Konkuk University, 120 Neungdong-ro, Gwangjin-gu, Seoul 05029, Republic of Korea; purple090@konkuk.ac.kr (B.S.); ppdh888@konkuk.ac.kr (D.P.); lsh8907@konkuk.ac.kr (S.L.); 2 Department of Veterinary Pathology, College of Veterinary Medicine, Konkuk University, 120 Neungdong-ro, Gwangjin-gu, Seoul 05029, Republic of Korea; kwjihoon@konkuk.ac.kr (J.K.); suhyunglee@konkuk.ac.kr (S.-H.L.); 3 Department of Veterinary Medical Imaging, College of Veterinary Medicine, Konkuk University, 120 Neungdong-ro, Gwangjin-gu, Seoul 05029, Republic of Korea; quieor@konkuk.ac.kr

**Keywords:** carcinoma of unknown primary origin, skeletal metastasis, multifocal osteolysis, poorly differentiated carcinoma, immunohistochemistry, hypercoagulability, systemic thrombosis, thromboelastography

## Abstract

Carcinoma of unknown primary origin (CUP) involving the skeletal system is rare in dogs and may closely mimic primary bone neoplasia or multicentric lymphoma, creating substantial diagnostic challenges. A 15-year-old spayed female Jindo-mix dog was referred for rapidly progressive tetralimb lameness and inability to stand following a 2-week history of worsening forelimb lameness. Computed tomography revealed aggressive multifocal osteolytic and osteoproliferative lesions involving the pelvis, distal femur, and lumbar vertebra, accompanied by generalized lymphadenopathy, raising differential diagnoses including osteosarcoma, lymphoma, and metastatic neoplasia. Serial CT examinations demonstrated rapid progression of the dominant left ischial lesion, which was considered most consistent with a primary bone neoplasm and was surgically excised for definitive diagnosis. MRI failed to identify clinically significant neurologic disease. Histopathologic examination of a surgically excised ischial lesion favored poorly differentiated epithelial malignancy, although lymphoma could not initially be excluded. During hospitalization, serial thromboelastography (TEG) and coagulation testing demonstrated a predominantly hypercoagulable profile despite antithrombotic therapy. Following acute clinical deterioration, contrast-enhanced CT identified thrombosis involving the splenic vein and caudal vena cava, pulmonary thromboembolism (PTE), and concurrent multi-organ infarction. Cytology of an enlarged peripheral lymph node suggested metastatic carcinoma, and postmortem immunohistochemistry supported epithelial differentiation, and in the absence of an identifiable primary tumor despite comprehensive diagnostic evaluation, the case was clinically classified as carcinoma of unknown primary origin. This case highlights an unusual diagnostic presentation of skeletal CUP mimicking osteosarcoma or lymphoma and complicated by fatal cancer-associated thrombosis. Rapidly progressive multifocal bone lesions accompanied by hypercoagulability should prompt consideration of metastatic carcinoma, including carcinoma of unknown origin, even in the absence of an identifiable primary tumor.

## Simple Summary

Carcinoma of unknown primary origin (CUP) is an uncommon metastatic cancer in dogs in which the primary tumor cannot be identified despite extensive diagnostic evaluation. Because clinical signs are usually determined by the location of metastases, CUP can closely resemble other diseases and be difficult to diagnose. In this case, a 15-year-old Jindo-mix dog developed rapidly progressive multifocal bone lesions that initially appeared consistent with primary bone cancer or multicentric lymphoma. Histopathology alone was insufficient for definitive diagnosis because of the tumor’s poor differentiation, and immunohistochemistry was required to establish epithelial differentiation and support the diagnosis of poorly differentiated carcinoma. During hospitalization, the dog also developed persistent hypercoagulability followed by extensive systemic thrombosis, resulting in a fatal outcome. This report highlights that metastatic carcinoma should be considered among the differential diagnoses for dogs with rapidly progressive multifocal skeletal lesions, even when no primary tumor can be identified. It also emphasizes the diagnostic value of immunohistochemistry and the importance of recognizing cancer-associated hypercoagulability as a potentially life-threatening complication.

## 1. Introduction

Primary bone tumors in dogs are predominantly malignant, with osteosarcoma accounting for approximately 67–87% of malignant primary bone tumors, making it the most common skeletal neoplasm [1]. However, multifocal skeletal lesions broaden the differential diagnosis to include metastatic neoplasia, lymphoma, multiple myeloma, and osteomyelitis, thereby complicating differentiation from primary skeletal neoplasia [1,2].

Carcinoma of unknown primary origin (CUP) is a metastatic epithelial malignancy in which the anatomical site of the primary tumor cannot be identified despite comprehensive clinical, imaging, and pathologic evaluation. In human medicine, CUP accounts for approximately 2.3–4.2% of all malignancies and is generally associated with aggressive biologic behavior and poor prognosis [3]. Although retrospective studies and sporadic case reports describing CUP in dogs have been reported, the veterinary literature remains limited and diagnostic approaches are not well established [4,5,6]. Diagnosis may be particularly challenging in poorly differentiated neoplasms, in which histopathologic findings alone may be inconclusive and immunohistochemistry (IHC) can aid tumor classification and diagnostic confirmation [4].

Clinical manifestations of CUP are highly variable and generally reflect the sites of metastatic involvement rather than the occult primary tumor [4]. When metastatic disease predominantly involves the skeletal system, differentiation from primary bone tumors can be particularly challenging in dogs without a prior diagnosis of malignancy, as aggressive skeletal lesions may initially raise suspicion for primary bone sarcoma [7].

We describe a canine case of carcinoma of unknown primary origin presenting as rapidly progressive multifocal skeletal lesions, in which the dominant left ischial lesion initially mimicked a primary bone tumor and prompted surgical excision for definitive diagnosis. The case emphasizes the diagnostic challenges posed by skeletal-predominant CUP, the value of immunohistochemistry for lineage determination in poorly differentiated neoplasms, and the fatal systemic thrombotic complications associated with malignancy-related hypercoagulability.

## 2. Case Description

A 15-year-old spayed female Jindo-mix dog weighing 20 kg was referred to the Konkuk Veterinary Medicine Teaching Hospital with a chief complaint of progressive tetralimb lameness and inability to stand. The patient had a 2-week history of forelimb lameness, which progressively worsened to involve the hindlimbs, ultimately resulting in non-ambulatory status at the time of referral.

Physical and neurologic examinations were partially limited because of the patient’s non-ambulatory status. General physical examination did not reveal any palpable masses or clinically significant peripheral lymphadenopathy. No overt pain response was consistently elicited during orthopedic examination, including palpation and manipulation of the pelvic limbs. Mild neurologic deficits were suspected on initial examination, including mildly decreased proprioceptive positioning and hopping responses in all limbs except the left forelimb. However, these abnormalities were subtle and lacked reproducibility, as repeat neurologic examination performed 4 days later revealed normal postural reactions in all limbs.

**Figure 1:**
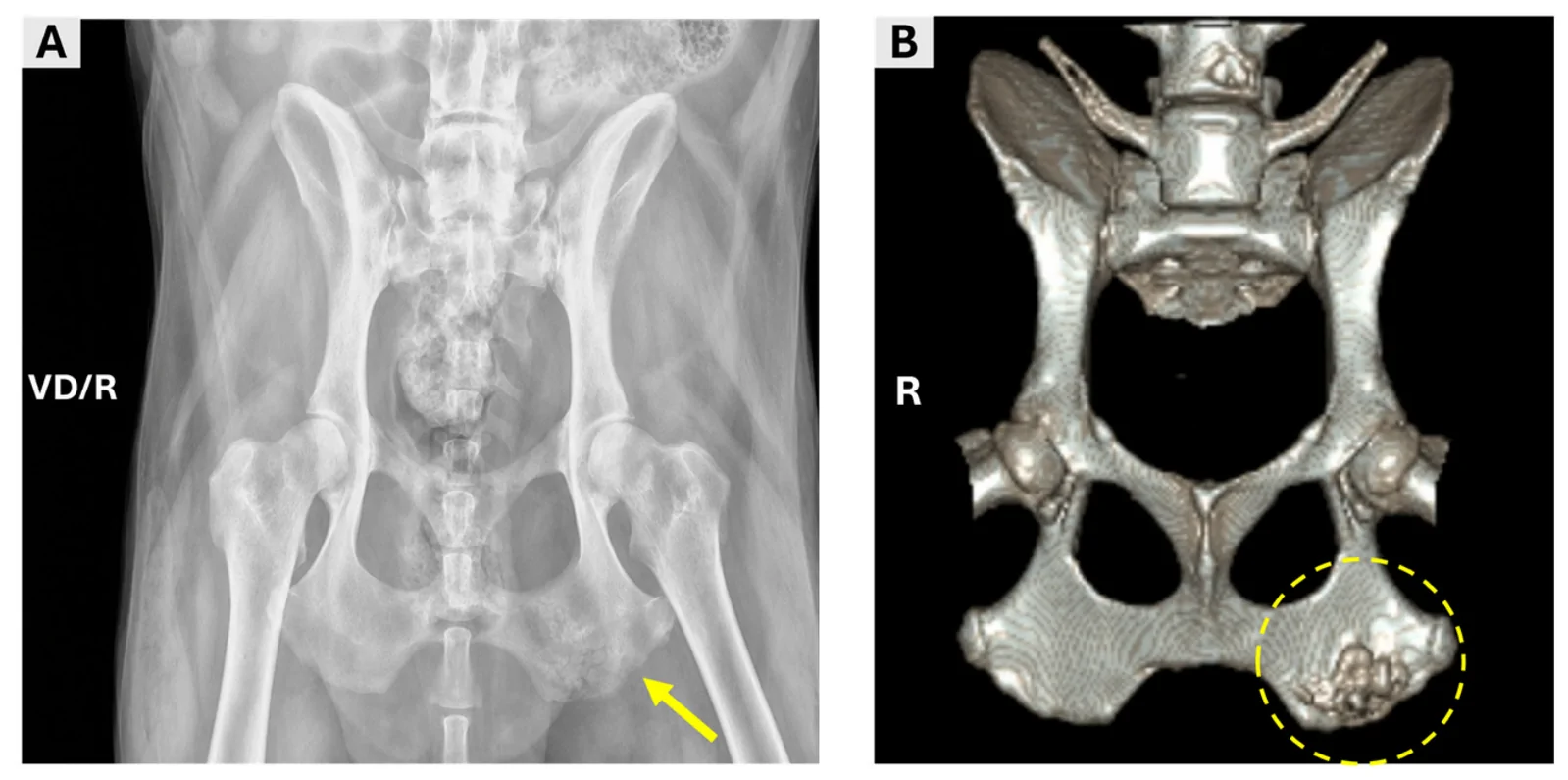
**Imaging findings at the second CT (pre-MRI) examination.** (**A**) Ventrodorsal pelvic radiograph demonstrating irregular periosteal proliferation and osteolysis involving the left ischial table (arrow). (**B**) Three-dimensional CT reconstruction of the pelvis (ventral view) demonstrating the dominant left ischial lesion (dashed circle).

**Figure 2:**
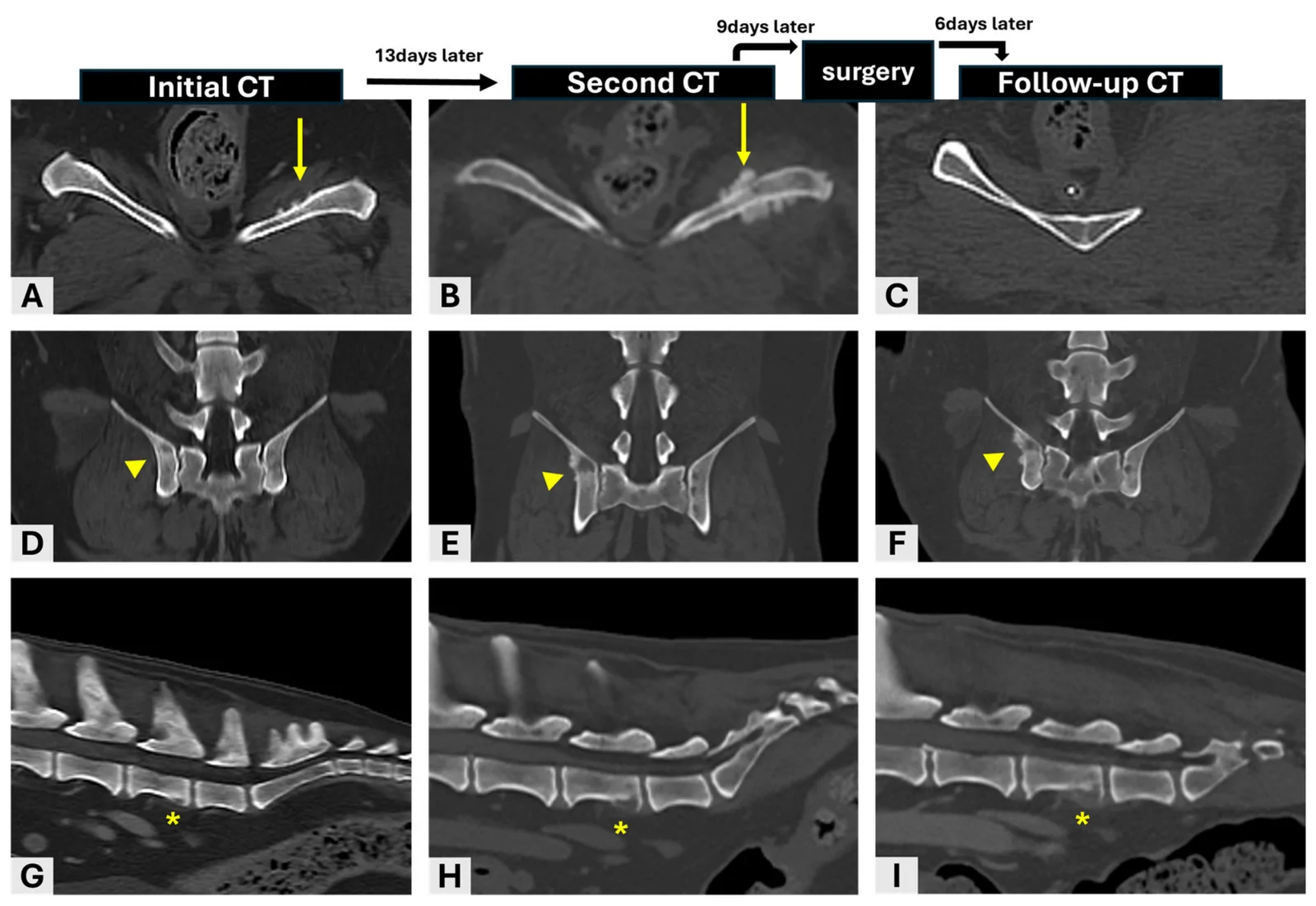
**Serial CT images obtained using a bone window (WL 500, WW 4000) demonstrating interval progression of multifocal skeletal lesions.** Images were obtained at **the initial CT examination**, **the second CT examination** performed 13 days later immediately before MRI, and **the follow-up CT examination** performed 6 days after partial ischiectomy. Axial images of the left ischial lesion are shown in (**A**–**C**), coronal images of the bilateral iliac lesions in (**D**–**F**), and sagittal images of the L6 vertebral lesion in (**G**–**I**). Progressive osteolysis and irregular periosteal proliferation are evident across the serial examinations (arrows, left ischial lesion; arrowheads, bilateral iliac lesions; asterisks, L6 vertebral lesion).

Survey pelvic radiographs obtained at presentation demonstrated an irregular periosteal reaction with associated osteolytic changes involving the left ischial table (Figure 1). Computed tomography (CT) had been performed at the referring hospital 13 days before referral for evaluation of bilateral forelimb lameness. Upon referral, review of the initial CT images at our institution identified aggressive osteolytic and osteoproliferative changes involving the left ischial table, characterized by cortical destruction and irregular periosteal proliferation (Figure 2A). Mild osteolytic and osteoproliferative changes were additionally identified in the right distal femur and bilateral ilium, indicating multifocal skeletal involvement at the time of presentation (Figure 2D). Generalized thoracic and abdominal lymphadenopathy with heterogeneous contrast enhancement was identified, involving the cranial mediastinal, tracheobronchial, periportal, and cranial and caudal mesenteric lymph nodes. Whole-body CT evaluation did not identify a discrete primary tumor in any of the examined organs or soft tissues. Based on these imaging findings, differential diagnoses for the osseous lesions included osteosarcoma and osteomyelitis. Furthermore, multicentric lymphoma, metastatic neoplasia, and systemic inflammatory disease were considered differential diagnoses.

Because neurologic disease could not be definitively excluded at presentation, magnetic resonance imaging (MRI) was performed. A pre-MRI CT (second CT) was concurrently performed to reassess the previously identified skeletal abnormalities and evaluate interval progression. MRI of the cervical spine demonstrated only mild left ventral spinal cord compression at the C2–C3 level, which was considered insufficient to explain the severity of the clinical signs. Compared with the prior CT examination performed 13 days earlier, the second CT demonstrated marked progression of the left ischial lesion, characterized by substantially worsening osteolysis and periosteal proliferation (Figure 2A,B). In addition, an osteolytic lesion involving the L6 vertebral body, which was subtle on the initial CT examination, was identified more clearly on the second CT, further supporting rapidly progressive multifocal skeletal disease (Figure 2G,H).

**Figure 3:**
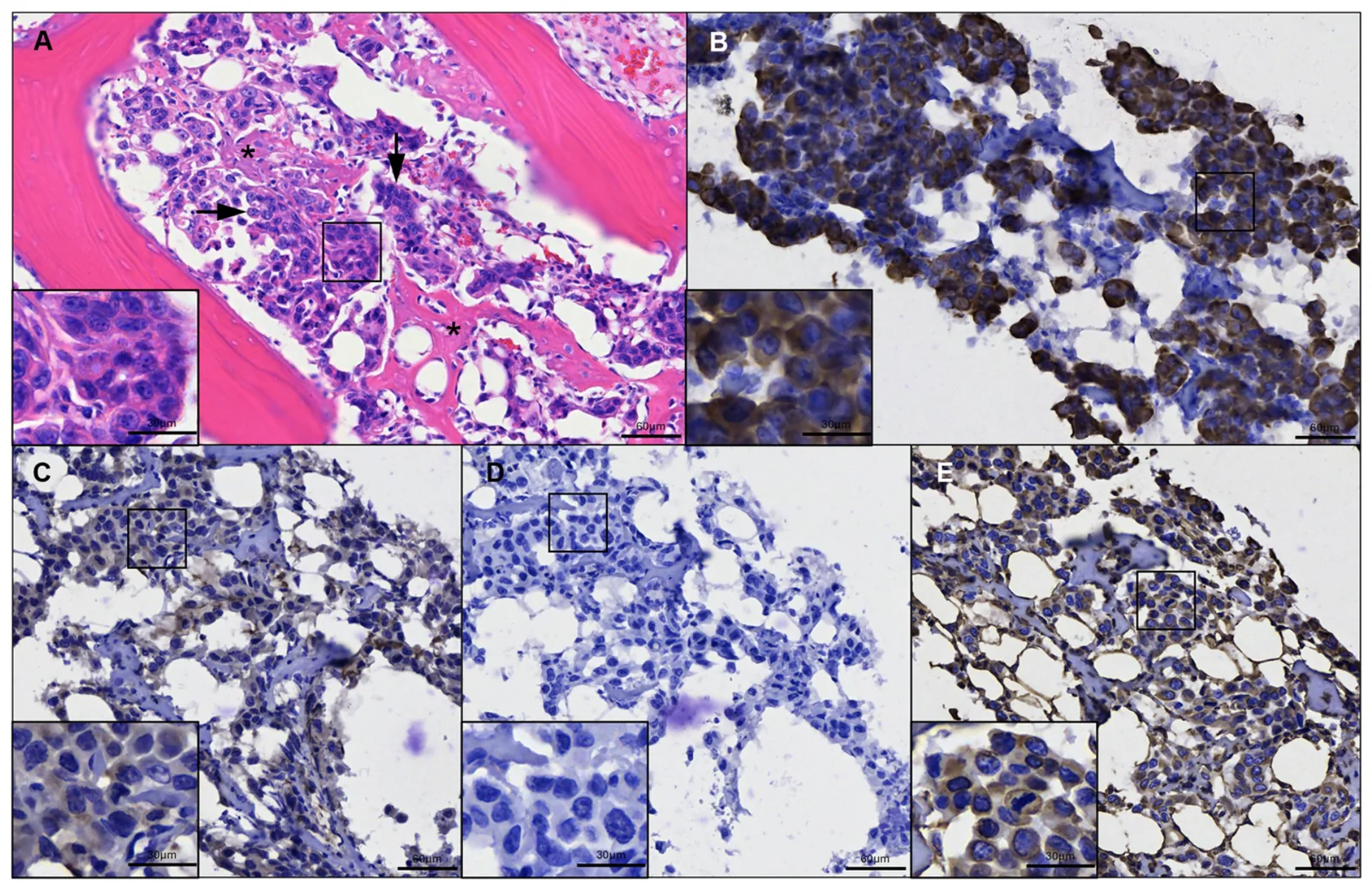
**Histopathologic and immunohistochemical findings of the left ischial lesion.** (**A**) Hematoxylin and eosin staining shows neoplastic cells infiltrating between bony trabeculae and osteoid (asterisks) and forming sheet-like aggregates (arrows). The neoplastic cells are polygonal to round, with moderate amounts of eosinophilic cytoplasm, marked anisokaryosis, nuclear pleomorphism, and prominent nucleoli (inset). (**B**) Immunohistochemistry for cytokeratin AE1/AE3 shows diffuse, strong cytoplasmic immunoreactivity in the neoplastic cells. (**C**–**E**) Immunohistochemistry shows that the neoplastic cells are negative for CD3 (**C**), PAX5 (**D**), and vimentin (**E**).

Partial ischiectomy of the dominant left ischial lesion was performed 9 days after the second CT examination for definitive diagnosis. Following induction of general anesthesia, the patient was positioned in right lateral recumbency, and a caudolateral approach to the left ischium was performed. The surrounding musculature was elevated as necessary to expose the affected ischium, while the sciatic nerve was identified and carefully protected. Following transection of the sacrotuberous ligament, osteotomies were performed using a Gigli wire saw and an oscillating saw, and the affected left ischium was subsequently removed en bloc. The surgical site was routinely closed in layers. Biopsy of additional skeletal lesions was not performed. A needle aspirate obtained from the excised osseous lesion was submitted for cytologic evaluation prior to decalcification. The excised specimen was subsequently decalcified and submitted for routine histopathologic examination (Figure 3). Cytologic findings were interpreted as suspicious for sarcoma, supporting an initial presumptive diagnosis of primary bone neoplasia.

Histopathological examination of the left ischial lesion revealed a diffuse neoplastic proliferation replacing the medullary cavity and infiltrating between bony trabeculae and osteoid (Figure 3A). The neoplastic cells were arranged in irregular clusters and sheet-like aggregates, with intercellular bridges observed between some tumor cells. The cells contained scant eosinophilic cytoplasm and exhibited marked anisocytosis and anisokaryosis. Cell borders varied from indistinct to relatively well defined, and the nuclei frequently contained prominent nucleoli (Figure 3A, inset). The presence of intercellular junctions and clustered growth patterns favored epithelial malignancy, with poorly differentiated carcinoma considered most likely. However, definitive classification remained challenging because of the markedly poor cellular differentiation. Although osteosarcoma was considered less likely, lymphoma could not be completely excluded in light of the generalized lymphadenopathy and concurrent clinical findings. Therefore, immunohistochemical (IHC) analysis was pursued for further characterization.

Postoperative recovery was initially satisfactory. Analgesia was maintained with continuous fentanyl infusion (2 μg/kg/h) followed by transdermal fentanyl patch (50 μg/h) during postoperative hospitalization. Assisted ambulation using a sling was required until postoperative day (POD) 2, and independent ambulation comparable to the preoperative status was regained by POD 3. Voluntary food intake was maintained until POD 5 but was lost from POD 6 onward.

During postoperative hospitalization, serial hematologic, biochemical, and coagulation monitoring demonstrated persistent systemic inflammation and abnormalities in coagulation parameters (Table 1). Serum C-reactive protein (CRP) concentrations transiently decreased after surgery but remained elevated throughout hospitalization. Serial thromboelastography (TEG) demonstrated a predominantly hypercoagulable state, with coagulation index (CI) values above the reference interval (−3 to 3) at most evaluated time points. Individual TEG parameters showed variable abnormalities, including shortened R and K values at selected time points. Maximum amplitude (MA), representing overall clot strength, generally remained within or near the upper limit of the reference interval (51–69 mm), with only selected measurements showing mild increases above the upper limit. Antithrombotic therapy with dalteparin (150 IU/kg SC q8h) was initiated, subsequently increased to 175 IU/kg SC q8h, with concurrent addition of clopidogrel (2 mg/kg PO q24h). D-dimer concentrations remained markedly elevated (>3000–10,000 ng/mL) throughout the hospitalization, and platelet counts progressively decreased, with thrombocytopenia developing during the postoperative period. The patient’s packed cell volume (PCV) initially decreased following surgery but recovered to 36.5% by postoperative day 3. However, the PCV subsequently declined to 25.9% by POD 6, necessitating packed red blood cell transfusion. Abdominal ultrasonography revealed a wedge-shaped hypoechoic region within the splenic parenchyma and echogenic intraluminal material within the splenic vein, raising suspicion for splenic infarction associated with splenic vein thrombosis. Consequently, contrast-enhanced follow-up CT was performed on postoperative day 6 for further evaluation.

**Figure 4:**
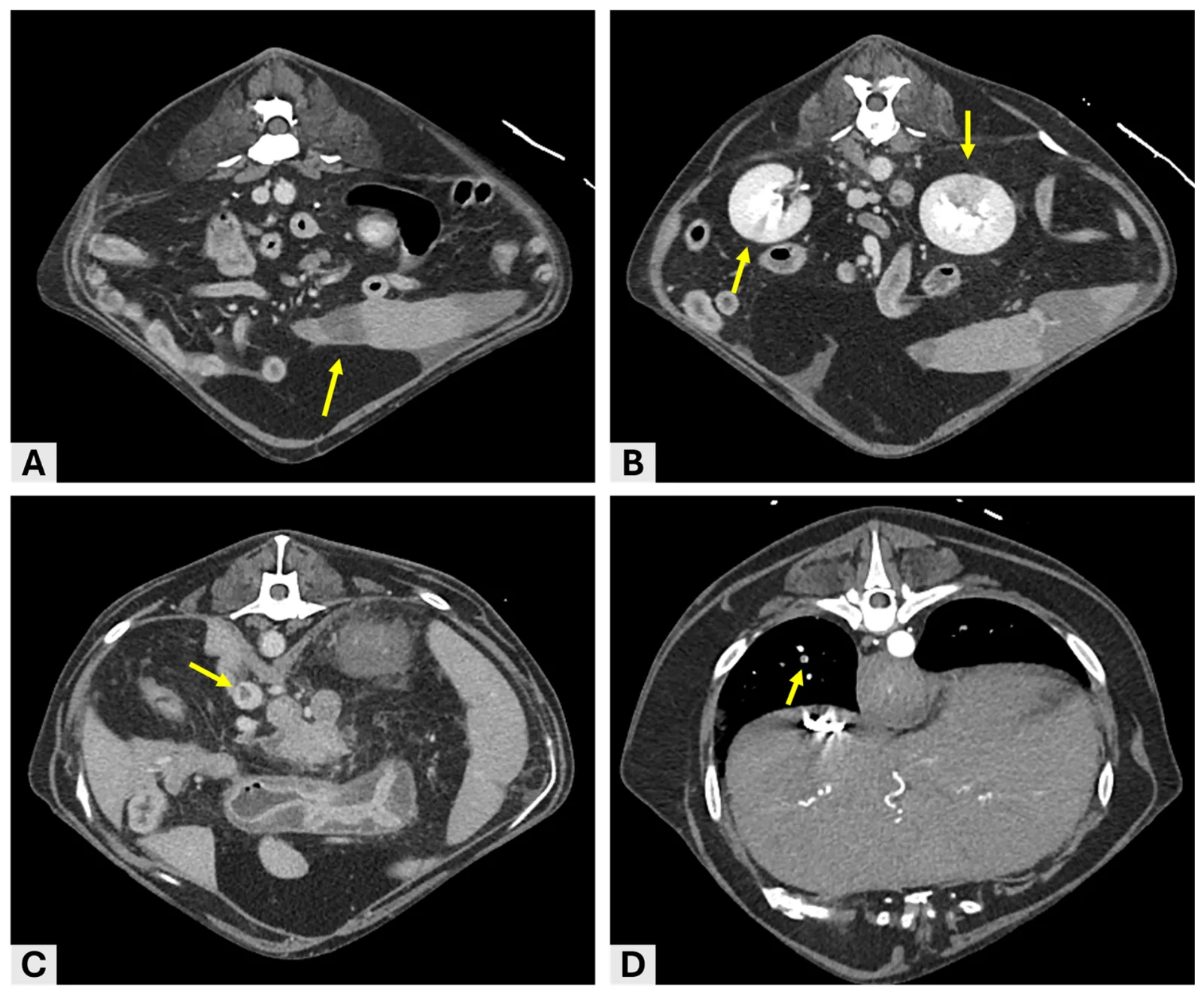
**Contrast-enhanced follow-up CT performed on postoperative day 6 demonstrating systemic thrombotic complications.** (**A**) Splenic infarction (arrow). (**B**) Bilateral renal infarctions (arrows). (**C**) Thrombosis of the caudal vena cava (arrow). (**D**) Pulmonary thromboembolism involving the right caudal lobar pulmonary artery (arrow).

Follow-up CT demonstrated multiple intraluminal filling defects consistent with thrombi within the splenic vein and caudal vena cava, as well as pulmonary thromboembolism (PTE) involving the bilateral caudal lobar pulmonary arteries (Figure 4C,D). In addition, multiple non-enhancing wedge-shaped hypoattenuating regions were identified within the spleen and bilateral renal cortices, consistent with splenic and bilateral renal infarctions (Figure 4A,B). Interval progression of the previously identified multifocal skeletal lesions and generalized lymphadenomegaly was also identified (Figure 2F,I). Cytologic evaluation of an enlarged left mandibular lymph node favored metastatic carcinoma, although concurrent lymphoma could not be excluded. Despite continued supportive and antithrombotic therapy for extensive systemic thrombosis, the patient progressively deteriorated and died on POD 10.

To further determine the lineage of neoplastic cells, immunohistochemistry for CD3, PAX5, vimentin, and cytokeratin AE1/AE3 was performed after the patient’s death. The neoplastic cells exhibited diffuse, strong cytoplasmic immunoreactivity for cytokeratin AE1/AE3 (Figure 3B), whereas no immunoreactivity was detected for CD3, PAX5, or vimentin (Figure 3C–E). This immunophenotype supported epithelial differentiation and did not support lymphoid or mesenchymal differentiation. Based on the combined histopathological and immunohistochemical findings, the lesion was diagnosed as a poorly differentiated carcinoma involving bone. In the absence of an identifiable primary tumor despite comprehensive diagnostic evaluation, the case was clinically classified as carcinoma of unknown primary origin (CUP).

## 3. Discussion

Given the mild and transient neurologic abnormalities and the rapid progression of multifocal skeletal lesions on serial CT, the progressive lameness and inability to stand were considered primarily attributable to multifocal skeletal disease and associated mechanical dysfunction rather than neurologic disease. However, determining the underlying cause of these skeletal lesions remained challenging because of their unusual multifocal distribution.

In dogs, aggressive bone lesions are most commonly associated with primary bone tumors, particularly osteosarcoma [2]. Accordingly, the rapidly progressive osteolysis and irregular periosteal proliferation observed in the dominant left ischial lesion, together with cytologic findings suspicious for sarcoma, reasonably supported an initial presumptive diagnosis of primary bone neoplasia. Similar radiographic and cytologic features have been reported in dogs with osteosarcoma [8]. However, clinical manifestations of carcinoma of unknown primary origin (CUP) are largely determined by the sites of metastatic involvement rather than the occult primary tumor itself [3,4]. Consequently, skeletal-predominant metastatic disease may closely mimic primary skeletal disorders both clinically and radiographically. Importantly, when multiple skeletal lesions are identified, the differential diagnosis broadens and includes multicentric bone neoplasia, lymphoma, multiple myeloma, metastatic neoplasia, and osteomyelitis. Although skeletal metastasis from carcinoma is uncommon in dogs, previous reports have documented extensive osseous involvement in canine metastatic carcinoma and CUP despite the absence of an identifiable primary tumor [4,6]. The present case further demonstrates that metastatic poorly differentiated carcinoma can closely resemble primary skeletal neoplasia when osseous lesions dominate the clinical presentation. Therefore, metastatic carcinoma, including CUP, should also be considered among the differential diagnoses for dogs presenting with rapidly progressive multifocal osseous lesions.

Multicentric osteosarcoma (MOSA) was considered an important differential diagnosis because of the multifocal skeletal distribution and aggressive osseous changes observed in this patient. Although the pathogenesis of MOSA remains controversial, previously proposed diagnostic considerations include multifocal skeletal lesions in the absence of widespread soft tissue metastasis and histologic similarity among affected skeletal sites [9,10]. In the present case, however, generalized thoracic and abdominal lymphadenopathy was already evident during initial staging, raising concern for disseminated systemic disease rather than a predominantly skeletal neoplastic process. Furthermore, histopathologic examination failed to support osteogenic differentiation, and immunohistochemistry ultimately confirmed metastatic poorly differentiated carcinoma. Collectively, these findings made primary skeletal neoplasia considerably less likely and prompted further consideration of alternative differential diagnoses, including lymphoma and multiple myeloma.

Multicentric lymphoma remained an important differential diagnosis because generalized thoracic and abdominal lymphadenopathy was identified during initial staging. In addition, lymphoma may occasionally involve the skeletal system and produce multifocal osseous lesions, further complicating differentiation from primary bone tumors and other metastatic diseases [11,12]. Furthermore, follow-up CT demonstrated progressive peripheral lymphadenomegaly, prompting cytologic evaluation of an enlarged left mandibular lymph node. Cytologic evaluation favored metastatic carcinoma, although the possibility of concurrent lymphoma could not be completely excluded based on cytology alone. Subsequent immunohistochemical characterization of the poorly differentiated neoplasm in the left ischial lesion demonstrated negative immunoreactivity for both CD3 and PAX5, providing no evidence of lymphoid differentiation in the sampled skeletal lesion. Multiple myeloma was also considered because of the multifocal osteolytic lesions observed on serial CT examinations [2]. However, additional diagnostic testing, including serum protein electrophoresis and evaluation for Bence-Jones proteinuria, failed to demonstrate evidence of monoclonal gammopathy. Furthermore, histopathologic examination of the excised lesion supported metastatic epithelial malignancy rather than a plasma cell neoplasm. Collectively, these findings made multiple myeloma unlikely. Osteomyelitis was considered a differential diagnosis during the initial evaluation; however, subsequent histopathologic and immunohistochemical findings were more consistent with metastatic carcinoma and did not support an inflammatory etiology.

Although primary epithelial neoplasms of bone are exceedingly rare in dogs [13,14], primary adamantinoma of a long bone has been historically reported in a dog [15]. Therefore, a primary skeletal epithelial origin could not be entirely excluded in the present case. However, the multifocal skeletal distribution and generalized lymphadenopathy were more consistent with metastatic carcinoma involving bone than with a primary skeletal epithelial neoplasm.

Overall, although the multifocal aggressive skeletal lesions initially raised consideration of several primary and systemic skeletal disorders, the subsequent diagnostic findings progressively favored an epithelial malignancy. In particular, the histopathologic and immunohistochemical findings of the ischial lesion supported epithelial differentiation, while the multifocal skeletal distribution and generalized lymphadenopathy favored metastatic disease over a primary skeletal neoplasm. Nevertheless, because only the left ischial lesion was histologically evaluated and no primary tumor was identified, the exact origin of the carcinoma could not be definitively established.

Partial ischiectomy was selected rather than needle biopsy because the dominant left ischial lesion demonstrated rapid interval progression with progressive osteolysis and periosteal proliferation, raising concern for impending pathologic fracture. In addition, excisional biopsy provided sufficient tissue for comprehensive histopathologic and immunohistochemical evaluation. Furthermore, early surgical intervention allowed partial resection of the affected ischium while the lesion remained amenable to a limited surgical approach, potentially avoiding the need for a more extensive pelvic resection.

Definitive diagnosis in this case relied heavily on immunohistochemical characterization of the neoplasm. Cytologic evaluation of the excised osseous lesion was initially interpreted as suspicious for sarcoma, whereas histopathologic examination following decalcification favored metastatic epithelial malignancy based on the presence of intercellular junctions and clustered growth patterns. However, definitive classification remained challenging because of the marked degree of cellular atypia and poor differentiation. In addition, lymphoma could not be completely excluded on the basis of histopathologic findings alone, particularly given the generalized lymphadenopathy identified on staging examinations. These findings highlight the diagnostic limitations of morphology alone when evaluating poorly differentiated neoplasms involving skeletal system. Immunohistochemistry ultimately resolved this diagnostic uncertainty. The neoplastic cells demonstrated diffuse positive immunoreactivity for cytokeratin, supporting epithelial differentiation, whereas CD3, PAX5, and vimentin were negative. These findings strongly supported epithelial differentiation while arguing against lymphoid and mesenchymal neoplasia. Similar diagnostic challenges have been described in canine CUP, in which poorly differentiated tumors may be difficult to classify using routine histopathology alone and frequently require ancillary techniques such as immunohistochemistry for definitive diagnosis [4]. The present case further emphasizes the importance of immunohistochemical evaluation in dogs presenting with aggressive multifocal skeletal lesions and poorly differentiated neoplasms, particularly when cytologic and histopathologic findings yield conflicting or inconclusive results.

A particularly notable feature of this case was the development of severe systemic thrombosis associated with persistent cancer-related hypercoagulability. Cancer-associated thrombosis (CAT) is a recognized complication of malignant neoplasia and is thought to result from complex interactions among tumor cells, platelets, endothelial cells, and the coagulation cascade, ultimately promoting thrombin generation and a prothrombotic state [16,17]. In human oncology, the association between malignancy and thrombosis has long been recognized and is classically described as Trousseau’s syndrome. This syndrome is now understood to involve multiple overlapping prothrombotic mechanisms, including tumor-derived coagulation factors, platelet activation, endothelial interactions, and fibrin formation [16]. In human CUP, multifocal osteolytic lesions are a recognized pattern of skeletal involvement, and thrombotic complications have also been reported in patients with malignancy of unknown primary origin [18,19]. The concurrent occurrence of rapidly progressive multifocal skeletal lesions and extensive systemic thrombosis in the present dog therefore resembles clinical manifestations described in human CUP, although this combination has rarely been documented as a single clinical presentation. Previous studies have demonstrated excessive activation of coagulation in dogs with malignant tumors, with laboratory evidence of hypercoagulability identified in a substantial proportion of affected patients [20]. More recently, prospective studies have further demonstrated that thromboembolic disease may be more prevalent in dogs with malignant neoplasia than previously recognized [21].

In the present case, serial thromboelastography (TEG) demonstrated a predominantly hypercoagulable profile, with the coagulation index (CI) exceeding the reference interval at most evaluated time points. Although individual TEG variables were variable throughout hospitalization and increased clot strength was not consistently demonstrated, the serial CI values supported the presence of hypercoagulability before CT confirmation of thrombosis. Persistently elevated D-dimer concentrations indicated ongoing fibrin formation and degradation and may have reflected the underlying thrombotic disease; however, given the limited specificity of D-dimer, other contributing factors, including the postoperative state, could not be excluded [21]. The progressive decline in platelet count may have reflected increased platelet consumption associated with thrombus formation, although other contributing factors could not be excluded. Conventional coagulation parameters, including PT, aPTT, fibrinogen concentration, and antithrombin activity, were not evaluated, limiting comprehensive characterization of the patient’s coagulation status. Nevertheless, contrast-enhanced CT provided direct evidence of extensive thrombosis involving the splenic vein, caudal vena cava, and pulmonary arteries, together with multiple organ infarctions. Dalteparin was initially selected as the primary anticoagulant based on previous veterinary recommendations for the management of venous thrombosis and PTE in dogs [22]. Following CT confirmation of extensive systemic thrombosis on postoperative day 6, the dalteparin dose was increased from 150 to 175 IU/kg SC q8h, and clopidogrel (2 mg/kg PO q24h) was concurrently added to provide additional inhibition of platelet activation. This dose adjustment was based on the CT-confirmed thrombotic disease rather than on TEG findings. Platelet function testing was not performed; therefore, the antiplatelet effect of clopidogrel was not directly assessed. No clinically apparent bleeding complications were observed during antithrombotic therapy. Collectively, these findings were considered consistent with a clinically relevant prothrombotic state in this patient. The extensive metastatic burden observed in this case may have contributed to the hypercoagulable state, consistent with previous reports demonstrating an increased risk of cancer-associated thrombosis in patients with metastatic disease [23]. Although malignancy was considered an important contributor, recent surgery and postoperative inflammation may also have promoted coagulation activation, and their relative contributions to the thrombotic events could not be determined. Despite antithrombotic therapy following recognition of hypercoagulability, thrombotic disease continued to progress, suggesting that malignancy-associated procoagulant activity may have contributed to the ongoing thrombotic process. To the authors’ knowledge, reports describing skeletal-predominant carcinoma of unknown primary origin complicated by fatal systemic thrombosis are exceedingly rare in veterinary medicine. This case expands the recognized clinical spectrum of canine CUP by demonstrating that disseminated skeletal metastatic disease may be accompanied by rapidly progressive systemic thrombotic complications and multiorgan infarction.

This report has several limitations. First, as a single case report, the findings cannot be generalized to all dogs with carcinoma of unknown primary origin (CUP). Despite comprehensive diagnostic evaluation, including repeated contrast-enhanced whole-body CT examinations and histopathologic assessment, the primary tumor could not be identified. The immunohistochemical panel used in this case was primarily intended to determine tumor lineage rather than to establish the tissue of origin. The absence of necropsy represents an important limitation of this case, as definitive localization of the primary site was not possible. A complete postmortem examination might have revealed a small or occult primary tumor that was undetectable during antemortem evaluation and could have provided additional information regarding the extent of metastatic disease and the cause of death. Histopathologic evaluation was limited to the surgically excised left ischial lesion, and the remaining skeletal lesions were not sampled; therefore, histopathologic confirmation of the remaining lesions was not available. Additional skeletal lesions were not sampled because of the multifocal disease distribution and concern regarding procedure-associated morbidity. In addition, although cytologic evaluation of the enlarged left mandibular lymph node favored metastatic carcinoma, concurrent lymphoma could not be definitively excluded because histopathologic or immunohistochemical evaluation of the lymph node was not performed. Finally, because skeletal-predominant CUP is exceedingly rare in veterinary medicine, its clinical spectrum, biological behavior, and optimal diagnostic and therapeutic strategies remain poorly characterized, highlighting the need for additional well-documented cases to better define its clinical characteristics and optimal management.

## 4. Conclusions

To the authors’ knowledge, this is one of the few reported canine cases of carcinoma of unknown primary origin presenting predominantly as rapidly progressive multifocal skeletal lesions and subsequently complicated by fatal systemic thrombosis. This case highlights the diagnostic challenges posed by skeletal-predominant metastatic carcinoma, underscores the value of immunohistochemistry for definitive diagnosis of poorly differentiated neoplasms, and emphasizes that persistent cancer-associated hypercoagulability may precede life-threatening systemic thrombosis in dogs with disseminated malignancy.

## Figures and Tables

**Table 1:** Serial coagulation parameters, inflammatory markers, and antithrombotic therapy during postoperative hospitalization.

Parameter	POD4	POD5	POD6	POD7	POD8	POD9	RI
**R** **(min)**	1.8	2.8	2.0	2.9	6.3	2.8	2–8
**K** **(min)**	1.2	1.0	0.9	1.5	2.5	1.3	1–3
**α-angle** **(** **°** **)**	76.4	74.1	76.7	68.0	55.4	62.2	55–78
**MA** **(mm)**	66.8	69.5	69.1	67.9	69.5	71.7	51–69
**CI**	4.5	4.2	4.8	3.2	−0.2	3.4	−3.0–3.0
**D-dimer** **(ng/mL)**	>10,000	4931.65	5028.95	7143.51	3014.25	3988.18	<250
**Platelet** **(×10** **^3^** **/uL)**	157	178	148	142	143	102	148–484
**CRP** **(mg/L)**	45.0	36.0	51.0	92.8	88.8	101.9	0–20
**Antithrombotic** **therapy**	Dalteparin 150 IU/kg SC q8h	Dalteparin 150 IU/kg SC q8h	Dalteparin 150 IU/kg SC q8h	Dalteparin 175 IU/kg SC q8h, Clopidogrel 2 mg/kg PO q24h	Dalteparin 175 IU/kg SC q8h, Clopidogrel 2 mg/kg PO q24h	Dalteparin 175 IU/kg SC q8h, Clopidogrel 2 mg/kg PO q24h	-

RI = reference interval; R = reaction time; K = clot formation time; α-angle = rate of clot formation; MA = maximum amplitude; CI = coagulation index; POD = postoperative day, CRP = C-reactive protein.

## Data Availability

The original contributions presented in this study are included in the article. Further inquiries can be directed to the corresponding author.

## Abbreviations

The following abbreviations are used in this manuscript:

CBC	Complete blood count
CRP	C-reactive protein
CT	Computed tomography
CUP	Carcinoma of unknown primary origin
FNA	Fine-needle aspiration
IHC	Immunohistochemistry
MRI	Magnetic resonance imaging
MM	Multiple myeloma
MOSA	Multicentric osteosarcoma
PAX5	Paired box protein 5
POD	Postoperative day
PTE	Pulmonary thromboembolism
TEG	Thromboelastography
CAT	Cancer-associated thrombosis
MA	Maximum amplitude
CI	Coagulation index
